# Expression of Toll-Like Receptors in the Animal Model of Bladder Outlet Obstruction

**DOI:** 10.1155/2020/6632359

**Published:** 2020-12-12

**Authors:** Grzegorz Niemczyk, Lukasz Fus, Katarzyna Czarzasta, Anika Jesion, Piotr Radziszewski, Barbara Gornicka, Agnieszka Cudnoch-Jedrzejewska

**Affiliations:** ^1^Department of Experimental and Clinical Physiology, Laboratory of Centre for Preclinical Research, Medical University of Warsaw, Pawinskiego 3c, 02-106 Warsaw, Poland; ^2^Department of Urology, Medical University of Warsaw, Lindleya 4, 02-013 Warsaw, Poland; ^3^Department of Pathology, Medical University of Warsaw, Pawinskiego 7, 02-106 Warsaw, Poland; ^4^Department of Applied Pharmacy, Medical University of Warsaw, Banacha 1, 02-097 Warsaw, Poland

## Abstract

**Introduction:**

Bladder outlet obstruction (BOO) occurs in more than 20 percent of the adult population and may lead to changes in the structure and function of the bladder. The main objective of the study was to evaluate the expression of Toll-like receptor 4 (TLR 4) and Toll-like receptor 9 (TLR 9) in the animal model of BOO as potential triggers of the inflammation phase in the bladder. In addition, the modulating effect of alpha-1 adrenergic antagonist (tamsulosin) on TLR 4 and TLR 9 expression and inflammatory markers was assessed. *Material and Methods*. Thirty-two male, 9-week-old Sprague Dawley rats were randomly divided into 4 groups: SOP—sham-operated rats with a placebo (water); SOB—sham-operated rats with an alpha-1 adrenergic antagonist; BOOP—rats with BOO and a placebo; and BOOB—rats with BOO and an alpha-1 adrenergic antagonist. The rats were given a placebo or alpha-1 adrenergic antagonist for 15 days. Next, urine and the bladder were collected from the rats for histopathological and biochemical study.

**Results:**

Histopathological analysis showed chronic inflammation without acute inflammation in the bladder. TLR 4 showed positive cytoplasmic reactivity in the urothelium and the smooth muscles of the bladder. TLR 9 showed positive cytoplasmic reactivity only in the urothelium. BOO caused an increase in TLR 4 and TLR 9 expression. Furthermore, treatment with an alpha-1 adrenergic antagonist had no significant effect on TLR 4 and TLR 9 expression in rats with BOO. BOO caused a significant increase in urine concentration of interleukin 6 (IL-6), while alpha-1 antagonist reduced the urine concentration of IL-6 and the concentration of interleukin 18 (IL-18).

**Conclusions:**

The results suggest the participation of TLR 4 and TLR 9 receptors in the induction of inflammation in the bladder, which is the first phase in the development of pathophysiological changes in BOO.

## 1. Introduction

Bladder outlet obstruction (BOO), according to the International Continence Society (2019), is the general term for every obstruction during voiding characterized by reduced urine flow rate with simultaneously increased detrusor pressure [1]. It was estimated in 2018 that BOO affected 21.8 percent of the global adult population, and it affects more often in men than women (24.7 percent vs. 18.9 percent) [2]. The most common etiology of BOO is benign prostatic obstruction caused by benign prostatic hyperplasia (BPH) [3].

BOO causes burdensome lower urinary tract symptoms (LUTS) and in the long term may lead to irreversible changes in the bladder's structure which affects its function [4]. In experimental studies, BOO leads to prolonged decrease in blood flow in the bladder's tissues and ischemia [5]. Cycles of ischemia and reperfusion cause the generation of free radicals and result in tissue injury, i.e., ischemia-reperfusion injury (I/R injury) which induces inflammation (the inflammatory phase) [6, 7]. This is followed by the compensation phase characterized by muscular hypertrophy which enables the bladder to generate high intravesical pressure against BOO [7]. Finally, if the obstruction is not relieved, a decompensation phase occurs which is characterized by tissue fibrosis and impaired bladder function, and this is not fully reversible [7, 8]. Although similar changes are observed, BOO has much slower dynamics and is less studied in humans [4].

Toll-like receptors (TLRs) are potential triggers of the inflammatory phase. They are part of the innate immune system and belong to the family of pattern recognition receptors recognizing pathogen-derived compounds [9]. After activation, TLRs induce a strong inflammatory response [9]. Apart from pathogens, they recognize endogenous molecules released from damaged or dying cells—damage-associated molecular patterns (DAMPs) [10]. Activation of TLRs by DAMPs may lead to the release of free radicals and contribute to I/R injury; however, their role in the pathophysiology of BOO has not yet been investigated [11, 12]. The involvement of TLR 4 and TLR 9 in I/R injury is well studied, and for this reason, they were chosen for further detailed analysis [12, 13]. Additionally, we analyzed concentration of interleukin 1*β* (IL-1*β*), interleukin 6 (IL-6), and interleukin 18 (IL-18) as they are involved in different urinary tract pathologies and may be an effect of activation of TLR [14–16].

Adrenergic alpha-1 receptor antagonists are first-line treatment of LUTS associated with BOO caused by the prostate [17]. On the other hand, TLRs and adrenergic alpha-1 receptor antagonists may act synergistically to induce inflammation by cross-talk phenomenon [18, 19].

The objective of this study was to evaluate the impact of BOO on the expression of TLR 4 and TLR 9 in the bladder in the animal model of bladder outlet obstruction. Additionally, the study investigated the modulating effect of the adrenergic alpha-1 receptor antagonist on TLR 4 and TLR 9 expression, as well as inflammation in the bladder (white blood cell infiltration), and concentration of proinflammatory interleukins (IL-1*β*, IL-6, and IL-18) in urine which could also serve as potential diagnostic and prognostic biomarkers of BOO.

## 2. Material and Methods

### 2.1. Animals

Thirty-two male, 9-week-old Sprague Dawley (SPRD) rats, 250–270 g body weight, were used in the study. All experimental procedures were approved by The Local Animal Research Ethics Committee (158/2016) and were consistent with Directive 2010/63/EU of the European Parliament and of the Council of 22 September 2010 on the protection of animals used for scientific purposes. The rats were kept under standard conditions (temperature 22°–25°C; humidity 40%–60%; 12-hour light-dark cycle). The rats had free access to food and water.

### 2.2. Experimental Design

The animals were randomly divided into 4 experimental groups, 8 rats in each.

Group I: SOP—rats that underwent a sham operation and received a placebo (water).

Group II: SOB—rats that underwent a sham operation and received an adrenergic alpha-1 receptor antagonist (tamsulosin hydrochloride; T1330; Sigma Aldrich).

Group III: BOOP—rats that underwent a bladder outlet obstruction operation and received a placebo (water).

Group IV: BOOB—rats that underwent a bladder outlet obstruction operation and received adrenergic an alpha-1 receptor antagonist (tamsulosin hydrochloride; T1330; Sigma Aldrich).

On day 0, all animals underwent either a sham operation or a BOO operation as described below.

### 2.3. Surgical Procedures

All procedures were performed under general anesthesia (Ketamine 10 mg/100 g body weight i.p.; Xylazine 1 mg/100 g body weight i.p.). All rats underwent suprapubic midline incision to expose the bladder and urethra as previously described in the literature [20]. In rats in the BOO groups (i.e., BOOP and BOOB), an 18G needle was placed parallel to the urethra and then ligated with Vicryl 4-0 (Ethicon). Finally, the needle was removed to create a partial BOO, and the incision was closed.

The procedure was identical in sham-operated groups (SOP and SOB); however, ligation of the urethra was not performed.

In total, 4 rats died after surgery (1 rat in the SOP group, 2 rats in the SOB group, and 1 rat in the BOOB group).

The animals were then placed in individual home cages and were given an analgesic (Buprenorphine chloride 3 *μ*g/100 g body weight i.p., twice daily for 2–3 days) and an antibiotic (Penicillin, Polfa 10,000 IU/100 g body weight i.m.).

### 2.4. Treatment

For 15 days, rats in groups SOB and BOOB received the adrenergic alpha-1 receptor antagonist tamsulosin dissolved in 1 ml of water at a dose of 1 mg/kg administered through a feeding tube. In the SOP and BOOP groups, the rats received a placebo (1 ml of water) per oral lavage. According to the literature, observation for 15 days was sufficient to notice structural changes in the bladder [7].

### 2.5. Urine Collection

On the fifteenth day of the experiment, the rats were placed for 24 hours (from 6 pm to 6 am) in metabolic cages to collect urine. The urine was centrifuged at 2600 × g for 15 minutes at 4°C and kept at –80°C until further biochemical studies were undertaken.

### 2.6. Body Weight Measurement and Tissue Harvesting

On day 15, the weight of the rats was measured, and immediately after that, the rats were anaesthetized (Ketamine 10 mg/100 g body weight i.p., Xylazine 1 mg/100 g body weight i.p.) and sacrificed by intraperitoneal injection of a lethal dose of 1 ml/kg body weight i.p. of pentobarbital (Biowet Puławy). The bladders were harvested *en bloc*, weighted (to confirm the animal model of BOO) [21], and fixed in 10% buffered formalin.

### 2.7. Histological Analysis

Fixed samples were embedded in paraffin, cut into 3 *μ*m sections, and stained routinely with hematoxylin and eosin for morphological examination. The slides were digitized with a Hamamatsu NanoZoomer 2.0-HT scanner, and subjectively evaluated at 20x magnification using NDP.view2 software. The degree of acute inflammation (represented by neutrophils) and of chronic inflammation (represented by macrophages, lymphocytes, and plasma cells) was determined by a semiquantitative scale (none—no immune cells; low—a few immune cells; medium—a moderate number of immune cells; high—a significant number of immune cells).

### 2.8. Immunohistochemistry

**Figure 1:**
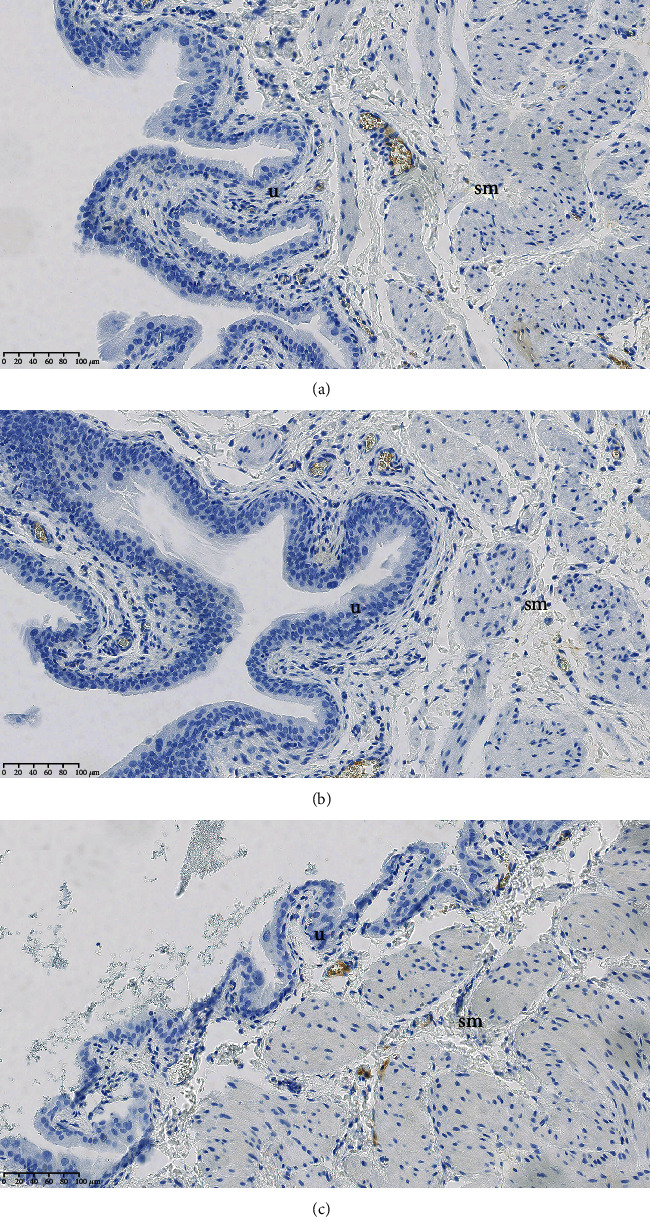
Negative controls of immunochemistry analysis. (a) Negative controls of TLR 4 immunostaining in the bladder with the primary antibodies omitted. (b) Negative controls of TLR 9 immunostaining in the bladder with the primary antibodies omitted. (c) Negative controls of alpha-1 adrenergic receptors immunostaining in the bladder with the primary antibodies omitted.

Bladder tissues were fixed in 10% buffered formalin and embedded in paraffin blocks. Immunohistochemical examinations were carried out on 3 *μ*m tissue sections deparrafinized in xylene and rehydrated in graded ethanol solutions. Heat-induced antigen retrieval was performed with EnVision FLEX Target Retrieval Solution, low pH (Dako Omnis). Endogenous peroxidase activity was blocked by incubation with EnVision FLEX Peroxidase-Blocking Reagent (Daco Omnis). After washing with PBS, slides with sections were incubated with 2.5% horse serum for 20 min to block the nonspecific antigen binding sites. Then, sections were incubated with primary antibodies against TLR 4 receptor (Anti-TLR4 antibody, ab22048, ABCAM), TLR 9 receptor (TLR9 Antibody, NBP2-24729, Novus Biologicals), and alpha-1 adrenergic receptor (Anti-alpha 1 Adrenergic Receptor antibody, ab3462, ABCAM) in dilutions in accordance to each manufacturers' recommendation. To detect primary antibodies, HRP-conjugated secondary antibodies were used (EnVision FLEX/HRP detection system, Dako Omnis). Finally, sections were counterstained with Mayer's hematoxylin, dehydrated, cleared in xylen, and coverslipped using mounting solution. To control the specificity and avoid nonspecific binding of primary antibodies, we evaluated tissues and cells (perivesical adipose tissue, nerve fibers and endothelium) present in each specimen that showed no reaction with tested antibodies (internal negative control). To avoid nonspecific binding of secondary antibody, additional negative controls were performed by omitting the primary antibodies (Figures 1(a)–1(c)). The immunohistochemically stained slides were scanned with a Hamamatsu NanoZoomer 2.0-HT scanner and viewed using NDP.view2 software and then subjectively evaluated.

The bladder sections were first assessed at low magnification (2x to 4x scanning magnification) to evaluate the general distribution of immunopositive cells. Next, three to five different fields were selected from the chosen section and viewed at 20x magnification. The distribution of immunopositive urothelial and smooth muscle cells for TLR4, TLR9, and alpha-1 adrenergic receptor for bladder samples in all of the groups was assessed semiquantitatively in a four class scoring system as none—negative reaction, low—weak positive reaction, medium —moderate positive reaction, and high—strong positive reaction.

### 2.9. Biochemical Analysis of Urine

Concentrations of IL-1*β*, IL-6, andIL-18 in urine were determined by enzyme-linked immunosorbent assay (ELISA). Studies were conducted in accordance with the recommendations of the manufacturers: Rat (IL-1*β*) Elisa Kit, SRB-T-83324, Sunred Biological Technology Co.; Rat Interleukin-6 ELISA Kit, E0079r, Wuhan EIAab Science Co.; Rat Interleukiin-18 ELISA Kit, E0064r, Wuhan EIAab Science Co.

### 2.10. Statistical Analysis

Comparisons of the mean values of each characteristic were made using one-way ANOVA with *post hoc* Bonferroni test for normal distributions and the ANOVA signed rank Kruskal-Wallis test with *post hoc* Dunn's test for nonparametric data and for data presented semiquantitatively. The differences were considered statistically significant if *p* < 0.05. The values presented in the text and in the figures are expressed as medians with the interquartile range. Statistical analysis was performed using Statistica software (version 13.3).

## 3. Results

### 3.1. Characteristics of Animals

The body weight of the rats was not significantly different between the examined groups (SOP group: 291 (275–306) g; SOB group: 317 (306–320) g; BOOP group: 312 (305–321) g; BOOB group: 319 (309–322) g). However, the bladder weight differed significantly between the groups of rats (SOP group: 90 (78–121) mg; SOB group: 152 (134–199) mg; BOOP group 164 (158–178) mg; BOOB group: 193 (175–249) mg (*p* < 0.01)) and was significantly higher in the BOOP group in comparison with the SOP group (*p* < 0.05).

### 3.2. Inflammatory State in the Bladder

**Figure 2:**
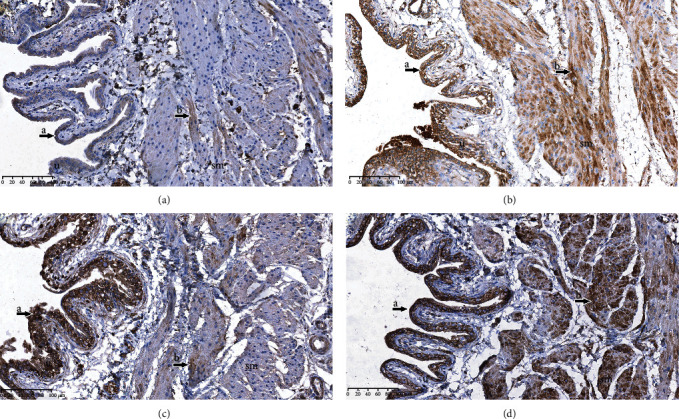
Inflammation in the bladder. (a) Rats that underwent sham operation and received a placebo (SOP group); (b) rats that underwent sham operation and received an alpha-1 adrenergic antagonist (SOB group); (c) rats that underwent bladder outlet obstruction operation and received a placebo (BOOP group); (d) rats that underwent bladder outlet obstruction operation and received an alpha-1 adrenergicantagonist (BOOB group). a: infiltration of mononuclear cells; u: urothelium; sm: smooth muscles.

In histopathological analysis, the bladder tissues were infiltrated with mononuclear cells, which is a characteristic of chronic inflammation, in the SOB, BOOP, and BOOB groups across the bladder layers (Figures 2(b)–2(d)). In the SOP group, mononuclear cells were not detected (Figure 2(a)). In semiquantitative analysis, mononuclear infiltration differed significantly between the groups (*p* < 0.01) and was more crucial in the BOOP group when compared to the SOP group (*p* < 0.05) (Table 1). Polynuclear cells, which are characteristic of acute inflammation, were not detected in any of the groups.

### 3.3. Immunochemistry Analysis of TLR 4 in the Bladder

**Figure 3:**
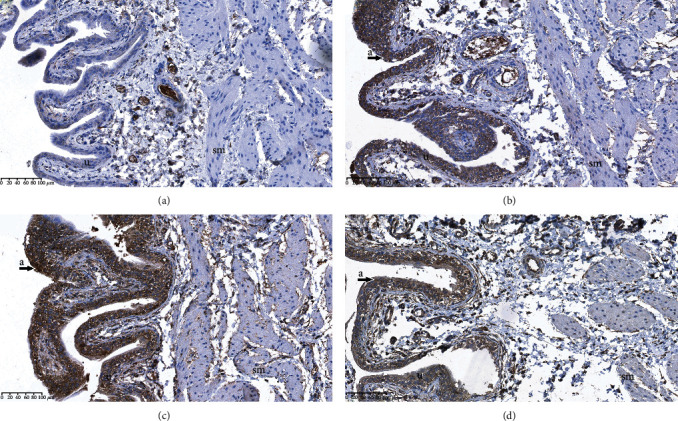
TLR 4 immunostaining in the bladder. (a) Rats that underwent sham operation and received a placebo (SOP group); (b) rats that underwent sham operation and received an alpha-1 adrenergic antagonist (SOB group); (c) rats that underwent bladder outlet obstruction operation and received a placebo (BOOP group); (d) rats that underwent bladder outlet obstruction operation and received an alpha-1 adrenergic antagonist (BOOB group). a: immunohistochemical cytoplasmic reactivity in urothelium; b: immunohistochemical cytoplasmic reactivity in smooth muscles; u: urothelium; sm: smooth muscles.

Immunohistochemical studies with TLR 4 showed positive cytoplasmic reactivity in the urothelium and the smooth muscles of the bladder in all groups of rats (Figures 3(a)–3(d)). Nuclear immunohistochemical reactivity was not detected in the urothelium nor in the smooth muscles of the bladder in any of the groups (Figures 3(a)–3(d)). Semiquantitative scoring intensities of TLR 4 showed significant differences both in the urothelium (*p* < 0.001) and in the smooth muscles (*p* < 0.01) between the examined groups of rats (Table 2). Expression of TLR 4 in the urothelium was significantly higher in the BOOP group in comparison with the SOP group (*p* < 0.001) and in the SOB group in comparison with the SOP group (*p* < 0.01). Similarly, expression of TLR 4 in the smooth muscles was significantly higher in the BOOP group in comparison with the SOP group (*p* < 0.05) and in the SOB group in comparison with the SOP group (*p* < 0.01) (Table 2).

### 3.4. Immunochemistry Analysis of TLR 9 in the Bladder

**Figure 4:**
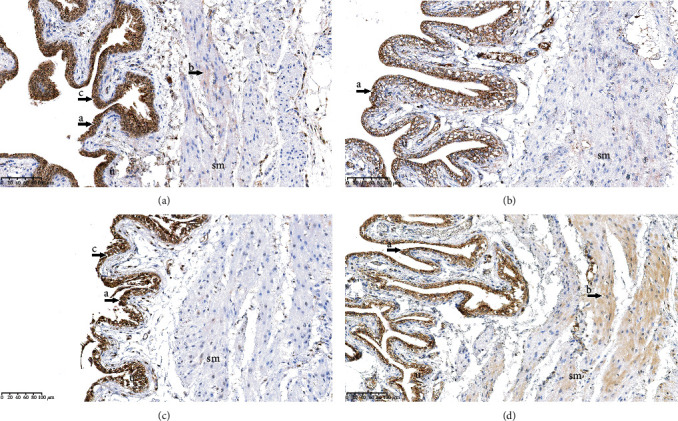
TLR 9 immunostaining in the bladder. (a) Rats that underwent sham operation and received a placebo (SOP group); (b) rats that underwent sham operation and received an alpha-1 adrenergic antagonist (SOB group); (c) rats that underwent bladder outlet obstruction operation and received a placebo (BOOP group); (d) rats that underwent bladder outlet obstruction operation and received an alpha-1 adrenergic antagonist (BOOB group). a: immunohistochemical cytoplasmic reactivity in urothelium; u: urothelium; sm: smooth muscles.

Immunohistochemistry showed positive cytoplasmic reactivity for TLR 9 in the urothelium in the SOB group, in the BOOP group, and in the BOOB group (Figures 4(b)–4(d)). Cytoplasmic reactivity in the smooth muscles was not detected as well as nuclear reactivity in both the urothelium and the smooth muscles (Figures 4(a)–4(d)). Semiquantitative scoring intensities of TLR 9 showed significant differences in the urothelium (*p* < 0.001) (Table 2). Additionally, TLR 9 expression in the urothelium of the bladder was significantly higher in the BOOP group in comparison with the SOP group (*p* < 0.001) and in the SOB group in comparison with the SOP group (*p* < 0.05) (Table 2). There were no significant differences in TLR 9 immunoreactivity in smooth muscles between the study groups of rats (Table 2).

### 3.5. Immunochemistry Analysis of Alpha-1 Adrenergic Receptors in the Bladder

**Figure 5:**
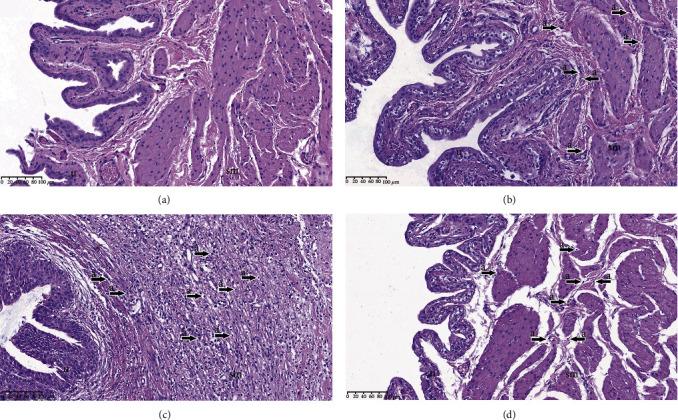
Alpha-1 adrenergic receptors immunostaining in the bladder. (a) Rats that underwent sham operation and received a placebo (SOP group); (b) rats that underwent sham operation and received an alpha-1 adrenergic antagonist (SOB group); (c) rats that underwent bladder outlet obstruction operation and received a placebo (BOOP group); (d) rats that underwent bladder outlet obstruction operation and received an alpha-1 adrenergic antagonist (BOOB group). a: immunohistochemical cytoplasmic reactivity in urothelium; b: immunohistochemical cytoplasmic reactivity in smooth muscles; c: immunohistochemical nuclear reactivity in urothelium; u: urothelium; sm: smooth muscles.

Immunohistochemistry showed positive cytoplasmic reaction for alpha-1 adrenergic receptors both in the urothelium and in the smooth muscles of the bladder in the SOP group and in the BOOP group (Figures 5(a) and 5(d)). However, cytoplasmic reactivity in the urothelium was detected only in the SOB group (Figure 5(b)). Additionally, nuclear reactivity was detected in the urothelium in the SOP group and in the BOOP group (Figures 5(a) and 5(c)).

### 3.6. Concentration of Interleukins in Urine

**Figure 6:**
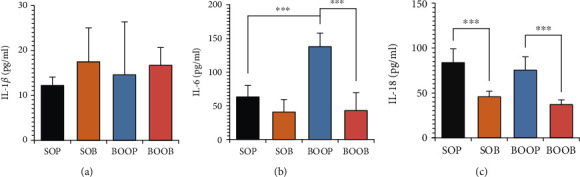
Concentration of interleukins in urine. (a) Concentration of interleukin 1*β* in urine. SOP: group that underwent sham operation and received a placebo; SOB: group that underwent sham operation and received an alpha-1 adrenergic antagonist; BOOP: group that underwent bladder outlet obstruction operation and received a placebo; BOOB: group that underwent bladder outlet obstruction operation and received an alpha-1 adrenergic antagonist; IL-1*β*: interleukin 1*β*. The results were evaluated by the Kruskal-Wallis test with *post hoc* Dunn's test. Medians with interquartile range are shown; (b) concentration of interleukin 6 in urine. IL-6: interleukin 6. Other abbreviations as in (a). The results were evaluated by the ANOVA test with *post hoc* Bonferroni test. Medians with interquartile range are shown; ^∗∗∗^*p* < 0.001. (c) Concentration of interleukin 18 in urine. IL-18: interleukin 18. Other abbreviations as in (a). The results were evaluated by the ANOVA test with *post hoc* Bonferroni test. Medians with interquartile range are shown; ^∗∗∗^*p* < 0.001.

Concentration of IL-1*β* in urine did not differ between the rat groups (Figure 6(a)). However, concentration of IL-6 in urine was significantly higher in the BOOP group than in both the SOP group (*p* < 0.001) and the BOOB group (*p* < 0.001) (Figure 6(b)). Urine concentration of IL-18 was significantly lower in the SOB group in comparison with the SOP group (*p* < 0.001) and in the BOOB group in comparison with the BOOP group (*p* < 0.001) (Figure 6(c)).

## 4. Discussion

In the present study, we showed that BOO is associated with chronic inflammation, upregulation of TLRs, and some inflammatory markers. Moreover, adrenergic alpha-1 receptor antagonists seem to counteract inflammation caused by BOO to some extent.

Animal model of BOO is confirmed by cystometry or by structural changes of the bladder (increased bladder mass or smooth muscles hypertrophy) [22]. We decided, based on previous studies, to confirm our animal model of BOO by analysis of the bladder mass, because functional aspects of BOO were not analyzed and cystometry, as invasive study could potentially influence results [23, 24]. In the present study, bladder weight was higher in the BOOP group in comparison with the SOP group. Moreover, there was an increasing trend of bladder weight in the BOOB group in comparison with SOB group, but the difference was not statistically significant. Perhaps this is connected with the relatively short observation time as, in the animal model of BOO, bladder mass progressively increased up to eighth week after the BOO operation [7].

In the histological analysis of bladder tissues, there were no immune cell characteristics for acute inflammation. This could be explained by the relative short time span of the acute phase of inflammation. In the study by de Almeida Prado et al., a transition from neutrophil (polynuclear) to macrophage (mononuclear) infiltration in the bladder tissues of Wistar rats with BOO was observed between the first and the fourteenth day [25]. Similarly, Liang et al. found macrophages infiltration in the bladder tissues of Sprague Dawley rats with BOO after 4 weeks [26]. Those studies confirm our finding that BOO causes chronic inflammation in the bladder tissues in animals with BOO. Interestingly, in SOB group, we found 2 cases of low chronic inflammation in comparison with none in SOP group. It could be a result of a random, more severe trauma during the operation in those cases, as well as, direct effect of alpha-1 adrenergic receptors blockers, but to our knowledge, there is no data in the literature confirming their inflammatory effect.

According to the available data, TLRs may play an important role in chronic inflammation [27]. In the present study, TLR 4 expression was elevated in the urothelium and in the smooth muscles in the group of the rats with BOO in comparison with sham-operated rats that received a placebo. Potentially, TLR 4 expression may be modulated by hypoxia as BOO results in reduced blood flow in the bladder [5]. In the study by Kim et al., 8-hour ischemia in murine macrophage cells resulted in the increased expression of TLR 4 at the mRNA and protein level and depended on the hypoxia-inducible factor 1*α* [28]. However, TLR 4 expression in human endothelial cells, which were incubated in hypoxic conditions for 72 hours (5% CO_2_ and 95% N_2_), decreased at the transcriptional and translational level [29].

Moreover, I/R injury may also play a role in modulating TLR 4 expression. In the study by Du et al., after 45 minutes of ischemia followed by 6 hours of reperfusion, the expression of mRNA of TLR 4 and its main transcription factor (NF-*κ*B) were elevated in the kidneys of Wistar rats [30]. Oxidative stress, as a result of I/R injury, must also be taken into consideration. Akther et al. proved that human leukocytes submitted to oxidative stress had higher TLR 4 expression at the mRNA and protein level [31]. Similarly, the mRNA of TLR 4 and NF-*κ*B were overexpressed in Sprague Dawley rats with acute kidney injury aggravated by oxidative stress [32]. Another important observation in the study by Akther et al. was that TLR 4 ligand lipopolysaccharide—that is a pathogen-derived compound—was responsible for the additional increase in receptor expression and depended on the TLR4/MyD88 signaling pathway [31]. Similarly, DAMP molecules that have an origin in I/R injury may also increase the expression of TLR 4 [33, 34].

TLR 9 expression was also elevated in the urothelium of the BOOP group in comparison with SOP group. Overexpression may be explained by the presence of stimulation by DNA containing CpG motifs, which is a ligand for TLR 9 [12]. This could have been derived not only from bacterial DNA but also from damaged mitochondria and could have functioned as a DAMP [12, 35]. In agreement with those presumptions, Ewaschuk et al. noted an increased expression of TLR 9 protein in colonic epithelial cells, which were exposed to pathogenic bacterial DNA [36]. I/R injury may also be an important factor in modulating TLR 9 expression. Ji et al. showed that 90-minute cerebral ischemia in C57BL/6 mice results in the upregulation of TLR 9 protein in microglia. The release of DAMPs was suggested by Ji et al. as the causative mechanism responsible for TLR 9 upregulation [37].

Interestingly, we found that tamsulosin increased the expression of TLR 4 and TLR 9 in the urothelium as well as expression of TLR 4 in the smooth muscles in the SOB group in comparison with the SOP group. This suggests a direct effect of the alpha-1 adrenergic receptor antagonist on cells in bladder tissues. However, there is not much data in the literature, and this requires further investigation.

In the present study, alpha-1 adrenergic receptors were expressed across the bladder and, moreover, in the same cells as the TLRs. According to data in the literature, a cross-talk phenomenon takes place between both receptors leading to their activation by a synergic effect. The simultaneous activation of alpha-1 adrenergic receptors and TLR 4 in human immune cells resulted in significantly higher IL-1*β* production than TLR 4 stimulation on its own [19]. In the study by Chen et al., beagles with low-grade chronic inflammation caused by intraperitoneal administration of lipopolysaccharide had a lower concentration of inflammatory markers (IL-6 and TNF*α*) after treatment with adrenergic alpha-1 receptor antagonist (doxazosin) [15].

Next, we analyzed proinflammatory interleukins associated with TLRs. In the animal models of BOO, IL-1*β* contributes to pathological changes in the bladder such as denervation and fibrosis [38, 39]. In the present study, IL-1*β* concentration in urine did not differ between the groups, and for this reason, it cannot be used as biomarker of BOO. However, it was proved that BOO caused increased protein expression of IL-1*β* in the bladder tissues of Sprague Dawley rats [24]. Similarly, in patients with BOO diagnosed in urodynamic study, upregulation of IL-1*β* signaling pathways was observed in biopsy samples taken from the bladders [40]. In the context of BOO, increased IL-1*β* expression may be a result of hypoxia as well as TLR 4 activation [14, 41]. On the other hand, urothelial cells are incapable of producing IL-1*β*, so this would explain lack of differences of its concentration in urine in cases where the urothelial layer was intact [42].

Interleukin 6 in urine was significantly upregulated in rats with BOO that received a placebo. This is in line with the study by Lin et al. in which BOO in Sprague Dawley rats caused increased expression of mRNA of IL-6 in bladder tissue [43]. Such an increase in IL-6 expression may be explained by the increased hydrostatic pressure caused by BOO, hypoxia, and also by the activation of TLRs [14, 26, 44]. In contrast with IL-1*β*, IL-6 may be excreted by urothelial cells which would explain the observed difference in its concentration in urine [42]. However, IL-6 in urine seems to be nonspecific and a characteristic for chronic inflammation, as its upregulation is observed in patients with interstitial cystitis [45]. Therefore, IL-6 may be helpful in detection inflammatory phase of BOO but cannot be used as a sole marker. Tamsulosin may have a local anti-inflammatory action as it downregulated IL-6 in urine in rats with BOO. According to the literature, alpha-1 adrenergic receptor antagonist (doxazosin) restricted IL-6 production induced by the stimulation of TLR 4 [15]. Downregulation of IL-6 without concomitant downregulation of TLR 4 expression in our study may be explained by interference with intracellular signaling pathways; however, different mechanisms independent of TLR may not be excluded.

The role of IL-18 in BOO has not yet been described in the literature; however, it was chosen for analysis as it takes part in other bladder conditions as bladder pain syndrome/interstitial cystitis. In the present study, BOO did not affect the concentration of interleukin 18 which could be explained by a lack of acute inflammation, and at this stage, it would not be useful in detection of BOO. On the other hand, IL-18 was affected by the administration of tamsulosin. IL-18 is excreted in a constitutive manner so an alpha-1 adrenergic receptor antagonist would seem to interfere with its production [46]. This is consistent with the study by Horstmann et al. in which human monocytes stimulated by phenylephrine (alpha-1 receptor agonist) resulted in the upregulation of IL-18 [47].

## 5. Conclusions

Our results suggest the participation of TLR 4 and TLR 9 receptors in the induction of inflammation in the bladder, which is the first phase in the development of pathophysiological changes in bladder outlet obstruction. Moreover, colocalization of TLR 4 and TLR 9 with alpha-1 adrenergic receptors in the urothelium and the smooth muscles enables a cross-talk phenomenon. Although blockade of alpha-1 adrenergic receptors does not significantly contribute to TLR 4 and TLR 9 expression in the bladders of Sprague Dawley rats, it reduces some inflammatory markers. Further studies on TLRs and an in-depth understanding of the pathophysiology of BOO may be helpful in the better management of the adverse effects of BOO on bladder structure and function and prevent irreversible changes. In the diagnostic aspect, IL-6, as a biomarker, may be helpful as an adjunct to prove BOO, but it needs further validation in human.

## Figures and Tables

**Table 1:** Semiquantitative analysis of the inflammation in the bladder.

Parameters	SOP (*n* = 7)	SOB (*n* = 6)	BOOP (*n* = 8)	BOOB (*n* = 7)	*p*
Acute inflammation	None (7/7)	None (6/6)	None (8/8)	None (7/7)	NS
Chronic inflammation	None (7/7)	None (4/6) Low (2/6)	Low ^∗^ (6/8) Medium (2/8)	Low (7/7)	<0.01

SOP: rats that underwent sham operation and received a placebo (water); SOB: rats that underwent sham operation and received an alpha-1 adrenergic antagonist (tamsulosin); BOOP: rats that underwent bladder outlet obstruction operation and received a placebo (water); BOOB: rats that underwent bladder outlet obstruction operation and received alpha-1 adrenergic antagonist (tamsulosin). The results were evaluated by the Kruskal Wallis test with post hoc Dunn's test. NS: nonsignificant. ^∗^Significant difference between the BOOP group and the SOP group; ^∗^*p* < 0.05.

**Table 2:** Semiquantitative scoring intensities of TLR 4 and TLR 9 immunolabeling in the bladder of the experimental groups.

Parameters	SOP (*n* = 7)	SOB (*n* = 6)	BOOP (*n* = 8)	BOOB (*n* = 7)	*p*
TLR 4 urothelium	1.86 ± 1.07	8.5 ± 1.22^&&^	9.0 ± 0^∗∗∗^	7.43 ± 3.05	<0.001
TLR 4 smooth muscles	2.38 ± 1.77	6.16 ± 1.6^&&^	5.25 ± 1.91^∗^	5.43 ± 2.44	<0.01
TLR 9 urothelium	1.38 ± 1.92	5.33 ± 1.03^&^	6.52 ± 2.27^∗∗∗^	6.00 ± 2.52	<0.001
TLR 9 smooth muscles	0.38 ± 0.74	0.5 ± 0.55	0.75 ± 0.46	0.86 ± 0.69	NS

SOP: rats that underwent sham operation and received a placebo (water); SOB: rats that underwent sham operation and received an alpha-1 adrenergic antagonist (tamsulosin); BOOP: rats that underwent bladder outlet obstruction operation and received a placebo (water); BOOB: rats that underwent bladder outlet obstruction operation and received alpha-1 adrenergic antagonist (tamsulosin). The results were evaluated by the Kruskal-Wallis test with post hoc Dunn's test. NS: nonsignificant. ^∗^Significant difference between the BOOP group and the SOP group; ^∗^*p* < 0.05, ^∗∗∗^*p* < 0.001. ^&^Significant difference between the SOB group and the SOP group; ^&^*p* < 0.05, ^&&^*p* < 0.01.

## Data Availability

The data used to support the findings of this study (precise characteristics of animals, concentrations of measured interleukins, and results of immunohistochemical analysis) are available from the corresponding author upon request.
